# Ultrasound-Based Quantification of Cartilage Damage After *In Vivo* Articulation With Metal Implants

**DOI:** 10.1177/19476035211063861

**Published:** 2021-12-11

**Authors:** Maria Pastrama, Janne Spierings, Pieter van Hugten, Keita Ito, Richard Lopata, Corrinus C. van Donkelaar

**Affiliations:** 1 Orthopaedic Biomechanics Group, Department of Biomedical Engineering, Eindhoven University of Technology, Eindhoven, The Netherlands; 2 Department of Orthopaedics, Maastricht UMC+, Maastricht, The Netherlands; 3 Cardiovascular Biomechanics Group, Photoacoustics and Ultrasound Laboratory Eindhoven, Department of Biomedical Engineering, Eindhoven University of Technology, Eindhoven, The Netherlands

**Keywords:** articular cartilage, surface roughness, focal knee resurfacing implant, ultrasound, Ultrasound Roughness Index

## Abstract

**Objective:**

This study aims to evaluate the applicability of the ultrasound roughness index (URI) for quantitative assessment of cartilage quality *ex vivo* (post-mortem), after 6 months of *in vivo* articulation with a Focal Knee Resurfacing Implant (FKRI).

**Design:**

Goats received a metal FKRI (*n = 8*) or sham surgery (*n = 8*) in the medial femoral condyles. After 6 months animals were sacrificed, tibial plateaus were stained with Indian ink, and macroscopic scoring of the plateaus was performed based on the ink staining. The URI was calculated from high-frequency ultrasound images at several sections, covering both areas that articulated with the implant and non-articulating areas. Cartilage quality at the most damaged medial location was evaluated with a Modified Mankin Score (MMS).

**Results:**

The URI was significantly higher in the FKRI-articulating than in the sham plateaus at medial articulating sections, but not at sections that were not in direct contact with the implant, for example, under the meniscus. The mean macroscopic score and MMS were significantly higher in the FKRI-articulating group than in the sham group ( P=0.035 , P<0.001 , respectively). Correlation coefficients between URI and macroscopic score were significant in medial areas that articulated with the implant. A significant correlation between URI and MMS was found at the most damaged medial location ( ρ=0.72,P=0.0024 ).

**Conclusions:**

This study demonstrates the potential of URI to evaluate cartilage roughness and altered surface morphology after *in vivo* articulation with a metal FKRI, rendering it a promising future tool for quantitative follow-up assessment of cartilage quality.

## Introduction

Focal cartilage defects (FCDs) can progress into further cartilage damage^1^ or osteoarthritis (OA).^2,3^ FCDs typically occur in young active people as a consequence of sport-related injuries, and in middle-aged people aged between 40 and 60 years.^2^ In this age group, in particular, a treatment gap exists for cartilage defect repair and early OA: while the regenerative capacity of articular cartilage is limited, thereby limiting the efficacy of regenerative approaches or microfracturing, these patients are too young to receive total joint replacement surgery.^4,5^ Focal knee resurfacing implants (FKRIs) are an emerging group of implants typically intended for the treatment of cartilage defects in middle-aged patients, which may bridge the treatment gap between cell-based regenerative therapies and total joint replacement. Most FKRIs investigated in animal models, or approved for clinical use, have an articulating surface made of metal, for example, cobalt-chrome,^6-16^ oxidized zirconium,^7,9^ or titanium.^17,18^ Although they showed good clinical outcomes in the treatment of isolated cartilage defects, it is not clear whether these implants prevent the progression of FCDs to OA in patients.^13^ Furthermore, animal studies have repeatedly shown that metal FKRIs cause damage to the opposing cartilage.^6-8,11,16^ While this damage is most likely a result of the mismatch between the mechanical properties of cartilage and the implant, most notably their stiffness, other factors such as the coefficient of friction between metal and cartilage—reported to be up to 10 times higher between CoCr and cartilage than between cartilage and cartilage^19-21^—and inaccurate implant positioning may also play a role.^7^ While considerable cartilage damage may occur in the cartilage opposing a metal implant irrespective of placement depth, the damage is significantly less when the implant is placed flush with the surrounding cartilage than when it is recessed or protruding.^7^ Importantly, a protruding and tilted metal implant was shown to be correlated to severe damage of the opposing tibial cartilage.^16^ To ensure accurate placement, a custom-made implanting device is recommended.^11,16^

In animal studies, the quality of the opposing (tibial) cartilage articulating with a metal FKRI used in a femoral defect is commonly investigated *ex vivo* after animal sacrifice. While some of these studies use various macroscopic scoring systems for such investigations,^7,9,16^ the golden standard remains histological scoring,^6-8,11,15^ which is a time-consuming technique. Furthermore, it cannot be used for patient follow-up after clinical interventions. As it is not possible to use Magnetic Resonance Imaging (MRI) in the presence of metal, *in vivo* monitoring of patients with metal FKRIs is limited to joint space narrowing as seen with radiography,^15,22^ an indirect measure of cartilage quality with low reproducibility, sensitivity, and specificity in detecting OA, disease progression, and cartilage damage features such as cartilage defects.^23^ Furthermore, radiography has limited performance in follow-up studies due to limited sensitivity to change over time and reproducibility issues when comparing time points in longitudinal studies, stemming from the dependence of joint space width on the positioning of the knee joint.^23,24^

Ultrasound (US) is a promising alternative tool able to provide direct internal soft tissue information, besides being safe, widely available, and cost-effective. In articular, the reflection of the cartilage surface primarily reflects the surface roughness, while the backscattering of the internal (micro)structure reflects the collagen fiber orientation and content and the chondrocytes.^25,26^ Previous research has shown promising results in detecting altered cartilage morphology with US, both *ex vivo* and *in vivo. In vivo*, US assessment of cartilage quality was shown to have very high sensitivity for femoral condylar cartilage damage, osteophytes, effusion/synovitis, and medial meniscal damage.^27^ In fact, US was shown to perform better than radiography in detecting osteophytes,^27,28^ at the same time providing more information on cartilage morphological changes.^28^ Very good agreement was found between US and arthroscopy, radiography, Magnetic Resonance Imaging (MRI), and intra-operative findings from TKA in detecting knee osteophytes and cartilage damage.^27-31^ Moreover, significant correlations were reported between qualitative *in vivo* US assessments, both arthroscopic^32^ and transcutaneous,^33^ and histological gradings.

Several US parameters and aspects from US images can be correlated with cartilage damage and clinical symptoms of OA in human subjects *in vivo*, such as decreased reflection coefficient, loss of interface sharpness, variation in internal echogenicity reflecting alterations in tissue composition, local thinning and consequent loss of cartilage thickness, and increased ultrasound roughness index (URI).^28,31,32^ The URI is a quantitative measure of the cartilage surface roughness and can describe morphological changes of the surface.^34,35^ Several *ex vivo* studies used the URI to assess cartilage degeneration in OA,^35-37^ mechanical or enzymatic degradation,^34^ following acute injury,^38^ with ovariectomy^39^ or after sliding shear.^40^ Furthermore, it was shown that the URI increases with deteriorating cartilage and is correlated with OA grade and histological scoring.^35-37^ However, to the best of our knowledge, the URI has not been used either *in vivo* or *ex vivo* to evaluate the quality of the opposing cartilage after articulation with metal FKRIs. Importantly, US measurements, including URI determination, may be done *in vivo* during arthroscopic surgery^32^ or potentially even transcutaneously in the future. As such, they may offer a possibility for quantitative determination of cartilage quality during diagnostic and follow-up after treatment. However, the sensitivity of this method to detect deterioration of cartilage must first be evaluated *ex vivo*.

The aim of this study is therefore to evaluate the applicability of US to detect changes in cartilage surface roughness *ex vivo* (post-mortem), after *in vivo* articulation with a metal FKRI, compared with articulation with intact femoral cartilage, by applying it to data of a 6-month goat study. If successful, the next step is to explore whether this method can be used noninvasively to ultimately apply it for follow-up assessment of cartilage quality in patients.

## Method

### Surgical Procedure

Medial femoral condyles of mature Dutch milk goats (aged 2-3 years, 60-80 kg) were bilaterally operated as part of another study. Approvals from the central commission for animal testing and local animal welfare committee of Maastricht University were obtained prior to this study (Approval Number: AVD107002016514).

**Figure 1.**
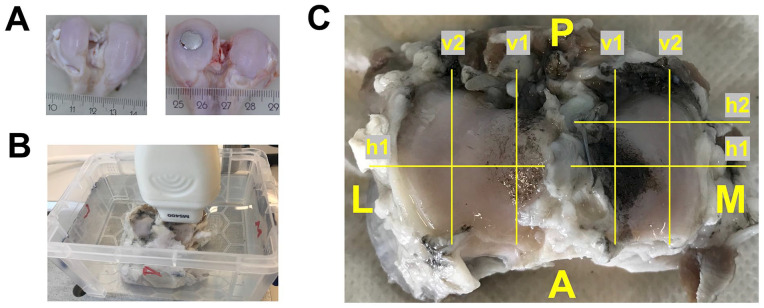
(A) Condyles without (left) and with (right) a medially placed metal implant, as retrieved after 6 months *in vivo*; (B) Ultrasound setup with a tibial plateau submerged in phosphate buffered saline and positioning of the ultrasound probe; (C) Schematic overview of the scan locations used in this study. The image in this figure is of a left tibial plateau that articulated with a metal implant.

Goats either received a metal FKRI (*n* = 8) or sham surgery (*n* = 8) in the medial femoral condyle of both left and right knee. Metal FKRIs consisted of a titanium (Ti6Al4V) stem and a polished cobalt–chromium–molybdenum (CoCrMo) articulating surface produced by machining (OHST Medizintechnik AG, Rathenow, Germany). They had a mushroom shape measuring 10.5 mm in height, with a 10-mm diameter top surface and a 6-mm diameter stem. The articulating top layer had a biconvex curvature with radii of 18 and 11 mm to match, respectively, the approximate sagittal and coronal curvatures of the goat knee. After anesthesia, the knees were opened, using an optimized medial parapatellar approach, to expose the medial femoral condyle.^41^ Implants were placed in the center of the condyle, using custom-made instrumentation as previously described by Jeuken *et al.*^42^ Briefly, a specifically designed 2.4 mm Kirschner-wire guide was first drilled to ensure perpendicular placement to the center of the condyle. Then, a custom-made depth-controller containing a cannulated drill allowed for incremental steps of drilling depth, aiming at a flush to slightly recessed implant position. After confirmation of the depth, using an undersized dummy implant, the actual implant was unpacked and press-fit into the defect by hammering. The wound was closed in layers using resorbable sutures. For sham surgeries, the medial femoral condyle was exposed as above, and the wound was closed without implant insertion. **Figure 1A** shows condyles as retrieved after 6 months with and without a metal implant in the medial side.

### Tissue Harvest and Storage

The goats were sacrificed 6 months post-surgery. After sacrifice, both knees were excised *en bloc*, using an oscillating saw, and subsequently dissected. The tibial plateaus were removed, stained with Indian ink (Royal Talens, The Netherlands), placed in neutral-buffered formalin formaldehyde 3.7% (v/v) in phosphate buffered saline (PBS)), for further processing and histology, and stored on a rocking platform at 4 °C for 2 weeks until US imaging. It has been previously shown that tissue fixation with formaldehyde does not produce significant changes in tissue acoustic parameters.^43^

### US Imaging

For image acquisition, the tibial plateaus were transferred to a tank filled with PBS at room temperature. US images were acquired using a high-frequency US system (Verasonics Vantage; Verasonics, Kirkland, WA, USA) connected to a 31.25 MHz linear transducer MS400 (FUJIFILM VisualSonics Inc., Bothell, WA, USA), with a bandwidth of 18-38 MHz. The US probe was mounted on a custom-made holder attached to a custom-made translation stage. For imaging, the probe was lowered into the tank and positioned perpendicular to the plateaus at a distance of a few millimeters to allow for imaging of the full cartilage thickness of the samples (**Fig. 1B**). The focus of the US system was manually set for each scan at the center of the region of interest. All 16 tibial plateaus (*n* = 8 articulating with a metal FKRI/metal group, *n* = 8 articulating with intact femoral condyles/sham group) were scanned at seven different locations, both Indian ink-stained and not ink-stained (damaged/not damaged), of which four were in the medial and three in the lateral compartment, resulting in a total of 112 scans (7 x 8 = 56 for the metal group and 7 x 8 = 56 for the sham group). **Figure 1C** gives a schematic overview of the scan locations. At each scan location, the URI was calculated, as described in the following section.

The scanned 2D planes in the medial compartment were in the medial-lateral (horizontal, *h*) direction as follows: (a) In the center of the compartment and of the damaged/ink-stained area that articulated with the implant (*Mh1*), and (b) approximately 0.5 cm posterior from Mh1, in an undamaged/not ink-stained area, previously covered by the meniscus (*Mh2*). In the anteroposterior (vertical, *v*) direction, the scan locations were as follows: (c) To the inside of the compartment, in the center of damaged/ink-stained area that articulated with the implant (*Mv1*), and (d) approximately 0.5 cm medial from Mv1, in an undamaged/not ink-stained area (*Mv2*), previously covered by the meniscus.

The scan 2D plane in the lateral compartment, in the medial-lateral (horizontal, *h*) direction, was as follows: (a) In the center of the compartment, corresponding to Mh1 (*Lh1*). In the anteroposterior direction, the scan locations were as follows: (b) To the inside of the compartment, corresponding to Mv1 (*Lv1*), and (c) approximately 0.5 cm medial from Lv1, corresponding to Mv2 (*Lv2*), previously covered by the meniscus.

### Quantification of the URI

**Figure 2.**
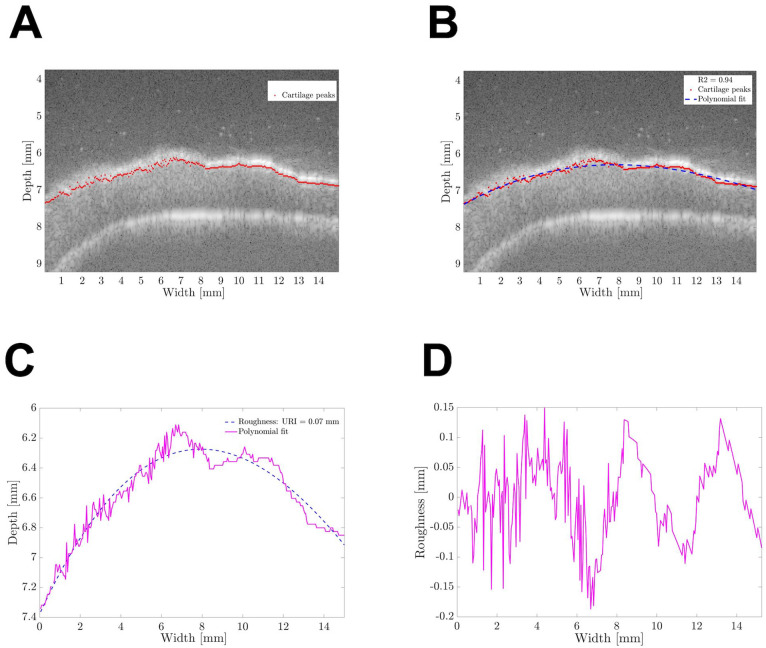
MATLAB-based algorithm for ultrasound roughness index determination. (A) Cartilage surface peaks detected by the algorithm are shown as red dots; (B) The polynomial estimate of the anatomical cartilage curvature is shown as a blue dashed line; (C) Superposition of the surface peaks and the polynomial without the original US image; and (D) The surface roughness profile determined after correcting for the anatomical cartilage curvature.

After US acquisition, B-mode images were reconstructed from the radio-frequent (RF) data and the data were further processed with MATLAB (2017b and 2019a; The MathWorks Inc., Natick, MA). The RF data were filtered using a low-pass Kaiser window filter (with an 8th order filter, a cutoff frequency of 1 kHz, and a sampling frequency of 31.25 MHz). Peaks in the enveloped RF data were detected per scan line in axial direction (**Fig. 2A**) and a third-order polynomial was fitted through the detected peaks, as an estimate of the anatomical curvature of the tibial plateau (**Figs. 2B and C**). After correcting for the anatomical curvature (**Fig. 2D**), the URI was calculated using Equation 1, adapted from Saarakkala *et al.*:^34^

(1) $$URI=\sqrt{\frac{1}{m}\sum_{i=1}^{m}d_{i}^{2}}$$

where m is number of scan lines and $d_{i}$ the height of the roughness peak after correcting for curvature in scan line i .

To test the repeatability and reproducibility of the URI quantification algorithm, eight random images were analyzed by two independent observers (one of them unexperienced). The experienced user quantified the URI using the in-house developed MATLAB algorithm twice for each image, while the inexperienced user quantified the URI of each image once. The intra-user variability for the URI determined for the eight images by the experienced user was, on average, 0.001%, and the inter-user variability was, on average, 0.006%. The Pearson correlation coefficients for intra- and inter-user variability were $R_{intra}^{2}\;=\;0.9936\;\left(P\;<\;0.0001\right)$ and $R_{inter}^{2}\;=\;0.9887\;\left(P\;<\;0.0001\right)$ , respectively.

### Macroscopic Feature Scoring

A macroscopic feature scoring system, adapted from Mastbergen *et al.*^44^ was created based on the appearance of the Indian ink staining in high-resolution digital photographs of the tibial plateaus (**Table 1**). The cartilage surface of the medial compartment was scored by seven independent observers blinded to the sample group (articulating with metal/sham). The average score from the seven observers was used as the representative score for each photograph. As the Indian ink stains only damaged areas, that is, those that had been in contact with the implant, the macroscopic scoring relates to cartilage quality only in these areas. The URI, however, is averaged over the damaged and undamaged areas that were included in an US scan line. Therefore, only scan lines that included damage, *i.e.*, scanning locations Mh1 and Mv1, correlated with the macroscopic score.

**Table 1.** Macroscopic Feature Scoring System for the Medial Plateau, Based on the Indian Ink Staining.

Smooth surface, no ink uptake	1
A few surface fibrillations, small gray or black stained area	2
Several surface fibrillations with a strong black stain	3
Many surface fibrillations and a large and strong black stain reaching to the center of the plateau	4
Damaged surface, a strong black stain, and bone visible under the cartilage	5

### Histology

After macroscopic scoring and US analysis, histology slides were prepared from medial tibial plateau regions, approximately corresponding to scanning location Mh1. A 3-mm coronal slab was cut from the tibial plateau using a band saw. The previously fixed specimens were dehydrated in increasing concentrations of ethanol in water up to 100% ethanol, followed by xylene and paraffin embedding. Sections of 5 μM were prepared, deparaffinized, and rehydrated using standard protocols. Proteoglycans were stained with Safranin-O (0.05%; Sigma-Aldrich) and counterstained with Fast Green (0.1%; Sigma-Aldrich). Stained sections were dehydrated and mounted in mounting medium (Histomount; Thermo Fisher Scientific, Waltham, MA). The sections were scanned using bright light microscopy at a magnification of 200x (M8 Microscope; Precipoint, Freising, Germany). Cartilage quality was evaluated according to an Osteoarthritis Research Society International (OARSI) histopathology initiative recommended Modified Mankin Score (MMS) by two blinded observers, and the average score was considered in further analyses.^45^

### Statistical Analysis

To check for normality, a Kolmogorov–Smirnov test was conducted. As most data sets were not normally distributed, Mann–Whitney U tests were performed to compare experimental groups. The Pearson correlation coefficient was calculated between the URI and the macroscopic feature score, and between the URI and MMS. Results are expressed as the M ± SD and significance is reported for *P* < 0.05. Statistical tests were performed using MATLAB (2020a; The MathWorks Inc., Natick, MA).

## Results

### URI

**Figure 3.**
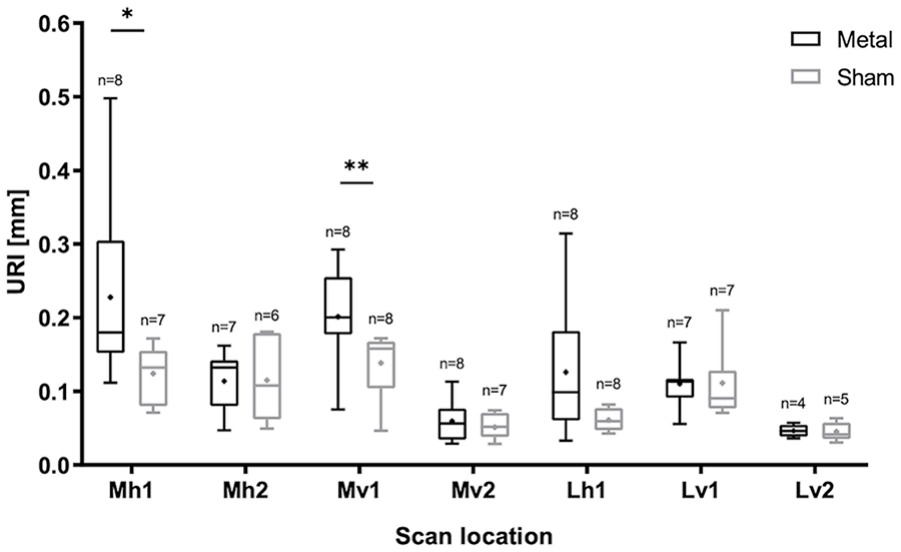
Boxplot of the 6-month follow-up data of tibial plateaus that articulated with metal implants or with condyles that received sham surgery for all scan locations described in **Figure 1C**. The average URI is indicated with “x” and the number of analyzed scans *n* is shown per subset. URI = Ultrasound Roughness Index. Significant differences *P* < 0.05 are indicated with *P < 0.001. **P < 0.01. ***P < 0.001.

For all scans, boxplots of the URI are shown per location and sample group (metal implant/sham) in **Figure 3**. A small number of scans was discarded because images could not be retrieved at a certain scan location due to the physical outline of the tibial plateau (*e.g.*, too steep), the fitted polynomial deviated significantly from the cartilage surface, or due to low image quality. The number of analyzed scans is shown in **Figure 3** for each data subset.

A significant difference in URI was found between the metal and sham group for scan locations Mh1 (*P* = 0.021) and Mv1 (*P* = 0.007). These locations correspond to the areas where the implant articulates with the tibial plateau and therefore where the most damage is expected and the strongest Indian ink staining was observed (**Fig. 1C**). The other medial areas scanned, Mh2 and Mv2, were largely covered by the meniscus and were not in contact with the implant. Therefore, at these locations, less or no damage is expected and there was no difference in URI found between the two groups. Similar outcomes were found at Lv1 and Lv2. At Lh1, the average URI of the metal group was slightly higher than that of the sham group, but the difference was not significant (*P* = 0.067). At most locations, the URI of the metal group was more variable than the sham, indicating more heterogeneity in the data.

### Correlation Between URI and Macroscopic Features

**Figure 4.**
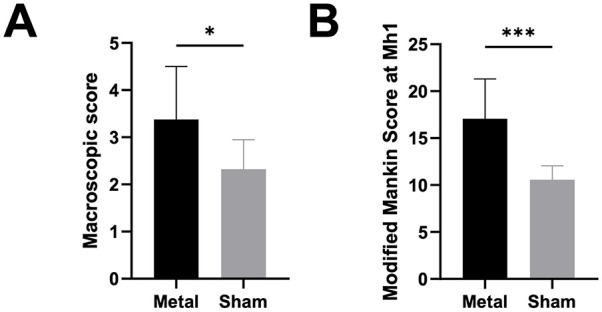
(A) Macroscopic feature score, and (B) Modified Mankin Score for the metal and sham groups (*M* ± *SD*). Significant differences *P* < 0.05 are indicated with *P < 0.001. **P < 0.01. ***P < 0.001.

The mean macroscopic score of the metal FKRI-articulating plateaus (3.38 ± 1.13) was significantly higher than that of the sham group (2.32 ± 0.62; *P* = 0.035; **Fig. 4A**).

When pooling all data from the metal and sham groups together, the correlation coefficients between URI and macroscopic score were significant in the damaged/ink-stained areas, that is, at scan locations Mh1 (ρ=0.75,P=0.0012) and Mv1 (ρ=0.52,P=0.038) . As expected, not damaged/not ink-stained locations showed no significant correlations.

The Pearson correlation coefficients between URI and macroscopic scoring at locations Mh1 and Mv1 in the metal group alone were 0.82(P=0.012) and 0.78(P=0.021) , respectively; correlations with the URI at all other locations in the metal group were not significant, and no significant correlations were found between URI and macroscopic score in the sham group alone.

### Correlation Between URI and MMS

The mean MMS at location Mh1 of the FKRI-articulating plateaus ( 17.06±4.23 ) was significantly higher than that of the sham group (10.56 ± 1.50; *P* < 0.001; **Fig. 4B**).

A significant correlation between URI and MMS for the pooled sham and metal data was found at this location (ρ = 0.72, *P* = 0.0024). The Pearson correlation coefficients between URI and MMS at location Mh1 in the metal group alone was ρ = 0.71, but did not reach statistical significance (*P* = 0.0502). As expected, no significant correlation was found between URI and MMS in the sham group alone because, in this group, both the URI and MMS values were homogeneous and low.

**Figure 5.**
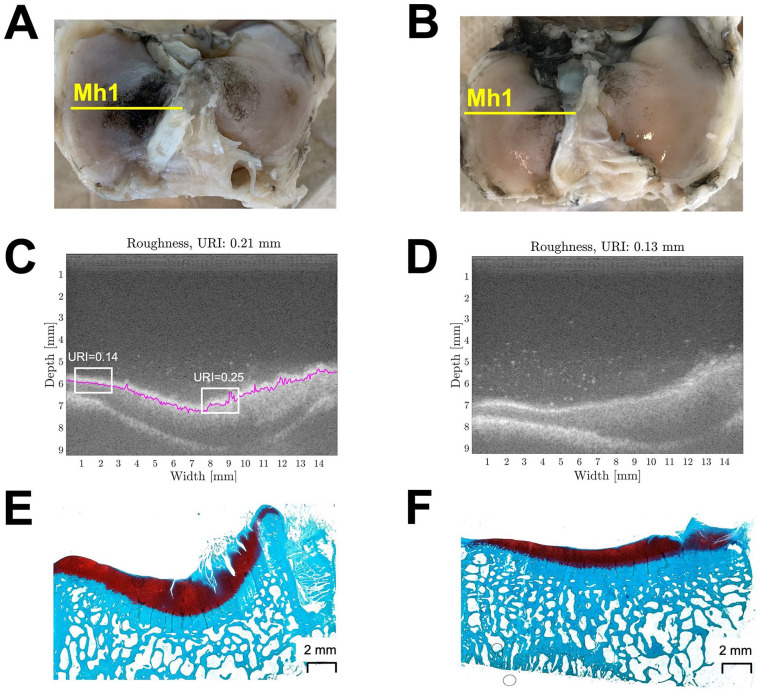
Example of a metal FKRI-articulating sample (Figures 5A, C, and E) and a sham sample (Figures 5B, D, and F). (A-B): Pictures indicating scanning location Mh1 used for the macroscopic Indian ink scoring; (C-D): Corresponding ultrasound image at location Mh1. The white boxes in C represent smooth and rough areas on the same metal-articulating sample with their corresponding URI; (E-F): Histology section at approximately location Mh1, used for the Modified Mankin scoring. FKRI = Focal Knee Resurfacing Implant; URI = Ultrasound Roughness Index.

Examples of macroscopic images, US images, and histology slides corresponding to location Mh1 of one representative metal FKRI-articulating tibial plateau and one representative sham plateau are shown in **Figure 5**. The tibial plateau articulating with the metal implant shows visible damage, strong Indian ink staining (macroscopic score 3.7, **Fig. 5A**), an URI of 0.21 (**Fig. 5C**), and an MMS of 15 (**Fig. 5E**). The white panels in **Figure 5C** show examples of a smooth and a rough area within the same metal-articulating sample with their corresponding URI. On the contrary, the sham sample appears smooth and presents almost no macroscopic damage (macroscopic score 2, **Fig. 5B**), the URI is 0.13 mm (**Fig. 5D**), and the MMS is 8.5 (**Fig. 5F**).

## Discussion

In this study, the feasibility of the URI to be used for quantitative assessment of cartilage quality after 6 months of *in vivo* articulation with a medially placed metal FKRI was investigated.

In the past, the URI was applied to assess cartilage degeneration *in vitro* with varying degrees of success. Saarakkala *et al.*^34^ found a significant increase in URI after mechanical degradation of bovine patellar cartilage by grinding the tissue surface with emery paper of different grit sizes. In the same study, the URI increased significantly with enzymatic digestion with collagenase but not trypsin and chondroitinase ABC. Wang *et al.*^39^ reported significant URI increases in condylar and tibial plateau cartilage of rats after ovariectomy. *In vivo*, Kaleva *et al.*^32^ found that URI measured arthroscopically could differentiate between intact and fibrillated cartilage. Conversely, Virén *et al.*^38^ reported no differences in URI between intact and acutely injured bovine cartilage samples through impact loading.

Here, we showed that URI can discriminate between tibial cartilage that articulated with a metal FKRI and tibial cartilage that articulated with intact condylar cartilage (sham group), which is a clinically relevant evaluation. Indeed, in the medial compartment, the URI was significantly higher in the metal group than in the sham group, and this was the case only at locations that were in direct contact with the implant (Mh1, Mv1). The significantly higher macroscopic score and MMS in the metal, compared with the sham group, at these locations demonstrate the higher amount of damage in the metal-articulating group. Our findings are in line with previous studies showing more cartilage degeneration in tibial plateaus in direct contact with a metal FKRI, compared with healthy knee joints.^7,9,11^ Conversely, as expected, in medial areas that were not in direct contact with the metal implant, that is, those not stained by the Indian ink and under the meniscus (Mh2, Mv2), there were no significant differences in URI between metal and sham plateaus. In these areas, and in lateral areas located under the meniscus (*e.g.*, Lv2), the URI is only determined by natural variability between animals and not by differences in treatment. The low *SD* of the URI in these regions indicates the good reproducibility of the method.

It is widely known that implant positioning is essential in such *in vivo* studies and a protruding implant can cause severe damage to the opposing cartilage.^7,11,16^ We used a custom-made device here, which ensured accurate positioning of the implant (no tilt and a flush or slightly recessed position).^42^ Implant position was always inspected visually after insertion during surgery, and macroscopically after sacrifice, when harvesting the condyles and tibial plateaus, confirming that the implants were not tilted or protruding. None of the implants showed any sign of loosening after 6 months *in vivo*.

In the lateral compartment, where no implant articulation took place, the URI was not different between the metal and sham groups at the vertical scan locations Lv1 and Lv2, that is, along the anatomical anteroposterior axis. Interestingly, however, at the horizontal location Lh1, that is, along the medio-lateral axis, the URI of the metal group was slightly higher than the sham although the difference was not statistically significant and the *SD* was very high, indicating that this area was damaged in some animals but not in others. This is in agreement with previous studies, showing more pronounced degeneration in the lateral plateaus of animals receiving a medial condylar cobalt–chrome implant compared with untreated controls, despite the lateral plateau not articulating directly against any implant.^7,9,46^ It has been previously suggested that this may be explained by altered joint homeostasis, and the fact that cartilage damage in the medial compartment may affect other areas in the joint through release of inflammatory cytokines or matrix-degrading proteases in the synovial fluid.^47,48^ Nevertheless, this does not explain the fact that increased URI was found in the lateral compartment only at Lh1, in the medio-lateral direction, but not at Lv1. An alternative explanation is that the animals altered their gait postoperatively, resulting in altered loading of the lateral compartment due to the surgical procedure and/or presence of the metal FKRI in the medial condyle, similar to what has been observed in patients with total knee arthroplasty.^49^ If this is the case, and damage is created along the articulation direction, that is, anteroposterior, then this damage may not be detected in US scans along this direction, but rather perpendicular to it. Although speculative, this may explain the higher lateral URI at Lh1 but not Lv1.

The macroscopic score correlated significantly with the pooled URI data from the two experimental groups at locations Mh1 and Mv1, representing the areas stained by Indian ink (**Fig. 1C**). When investigating individual, group-specific correlations between URI and macroscopic score at Mh1 and Mv1, it becomes apparent that the overall pooled correlation is driven by the correlation in the metal FKRI group, 0.82 (P=0.012) and 0.78 (P=0.021) , respectively, when the contribution of damaged tissue becomes more prominent in the evaluation, whereas no significant correlations were found at any location between URI and macroscopic score in the sham group alone. This suggests that the URI may be more accurate in predicting higher amounts of damage, while having reduced sensitivity to very low amounts of damage, such as that created by cartilage articulation in the sham samples. Mild cartilage degeneration in healthy or unoperated knees of sheep^16^ and Dutch milk goats of similar age as the ones used in our study^9^ has been previously reported. This is most likely due to the tendency of this species to spontaneously develop OA or OA-like changes as early as at 2 years of age.^45^

A significant correlation was found between URI and MMS (ρ=0.72,P=0.0024) in the pooled data. However, when analyzing the individual group correlations between URI and MMS, the Pearson correlation coefficient of the metal group was ρ=0.71 , but the *P* value was slightly above the significance level ( P=0.0502 ) and no significant correlation was found between URI and MMS in the sham group alone. As in the case of the macroscopic score, it appears that URI is more accurate in predicting more severely damaged samples than samples with little to no damage. These outcomes are in line with previous research, which found a correlation of 0.43 between Mankin score and URI of human condylar cartilage *in vitro*, although it did not reach significance.^37^ Furthermore, Niu *et al.*^35^ found differences in URI between healthy and OA rabbit cartilage samples. However, these differences were significant only between samples with OA OARSI Grade 3 (*more damaged*) and 0/1 (*intact/less damaged*), but not between samples with OARSI Grades 1 and 2. Mansour *et al.*^40^ used a three-level histological scoring of sliding-shear induced damage in cartilage and reported that URI only predicted the histology results if the sample was completely destroyed.

Indian ink staining and Mankin scores provide one damage grade for the entire joint or histological slice, respectively, and the URI is averaged over the whole scanning line including affected and unaffected areas. Only scanning locations Mh1 and Mv1 included the damaged area, which explains why the average URI correlated with the macroscopic score only in these lines. In the case of the Mankin score, it was determined in a slice corresponding to scanning line Mh1 and therefore correlated with the URI at this location. If only the affected parts of the scanning line had been used and unaffected areas had been excluded, the sensitivity of the method would have been even higher. This is illustrated in **Figure 5C**, where URI = 0.25 corresponds with an area that is stained with Indian ink in **Figure 5A** and clearly fibrillated in **Figure 5E**, whereas the URI = 0.14 area corresponds with an area that is not stained in **Figure 5A** and appears histologically healthy in **Figure 5E**.

A limitation of this study is the matching of histology and US scan locations. Although all histology and US images were visually checked to ensure there was a match in slice geometry and distinctive features, mismatches may result in disagreement between URI and MMS. In the future, such uncertainties may be addressed by performing a higher number of equally spaced histology slices and US scans or adding surface markers to the scanned locations.

URI provides a straightforward way to quantify the degree of damage. Such an objective measure would be of advantage in clinical applications and for research purposes, especially when it can be used in animal follow-up studies. While developing a noninvasive, transcutaneous device is the ultimate long-term goal, there are still many technological hurdles to overcome, stemming from the attenuation of US waves in the different knee structures that must be penetrated before reaching the cartilage. Integration of US imaging using a miniaturized probe in an arthroscopic device is a more feasible development for the near future and may be used together with URI in follow-up animal studies or in clinical cases where arthroscopy is performed anyway.

## Conclusion

We showed that *in vivo* articulation with metal FKRIs can lead to the formation of damage in the opposing tibial cartilage, and that high-frequency US measurements can reflect changes in the surface roughness index of cartilage, thereby distinguishing between damaged and undamaged samples. This study therefore demonstrates the potential of URI to evaluate cartilage roughness and altered surface morphology, rendering it a promising future approach for quantitative follow-up assessment of cartilage quality.
